# Alpha- and Gamma-Tocopherol and Telomere Length in 5768 US Men and Women: A NHANES Study

**DOI:** 10.3390/nu9060601

**Published:** 2017-06-13

**Authors:** Larry A. Tucker

**Affiliations:** Department of Exercise Sciences, Brigham Young University, Provo, UT 84602, USA; tucker@byu.edu

**Keywords:** cell aging, antioxidant, vitamin E, DNA

## Abstract

Antioxidants have a number of potential health benefits. The present investigation was designed to determine the relationship between serum alpha- and gamma-tocopherol levels (powerful antioxidants), and leukocyte telomere length (a biomarker of biological aging). A cross-sectional design was employed to study 5768 adults from the National Health and Nutrition Examination Survey (NHANES). DNA was obtained via blood samples. Telomere length was assessed using the quantitative polymerase chain reaction method. Serum concentrations of alpha- and gamma-tocopherol were measured using high performance liquid chromatography (HPLC). Results showed that for each one-year increase in age, telomeres were 15.6 base pairs shorter (*F* = 410.4, *p* < 0.0001). After adjusting for differences in the demographic covariates, for each µg/dL higher level of gamma-tocopherol, telomeres were 0.33 base pairs shorter (*F* = 7.1, *p* = 0.0126). Telomeres were approximately 1 year shorter (15.6 base pairs) for each increment of 47.3 to 55.7 µg/dL of gamma-tocopherol in the blood, depending on the variables controlled. Adults at the 75th percentile of gamma-tocopherol had 2.8–3.4 years greater cellular aging than those at the 25th percentile, depending on the covariates in the model. However, alpha-tocopherol was not related to telomere length. Evidently, gamma-tocopherol levels, but not alpha-tocopherol, account for meaningful increases in biological aging.

## 1. Introduction

Vitamin E is an essential nutrient and a powerful antioxidant. It is a fat-soluble vitamin that occurs naturally in eight forms. Vitamin E can be divided into two principal classes: tocopherols and tocotrienols. These can be further categorized into slightly different compounds, known as alpha, beta, delta, and gamma [1].

Many claims have been made about the potential of vitamin E to improve health and prevent disease because it is a chain-breaking antioxidant that prevents free radical reactions and lipid peroxidation. The most abundant and biologically active form of vitamin E is alpha-tocopherol [2]. Although alpha- and gamma-tocopherol differ by only one methyl group, alpha- is the only form of vitamin E considered necessary to satisfy human nutrition needs [3]. However, unlike alpha-tocopherol, gamma- also counters reactive nitrogen species [4]. Moreover, some scientists indicate that the antioxidant characteristics of gamma-tocopherol may actually exceed those of alpha-tocopherol [5]. In the US, blood levels of alpha-tocopherol are about five times higher than gamma-levels [6].

Although alpha- and gamma-tocopherol differ only slightly in molecular structure, some studies indicate that they have different consequences on the body [2,7,8,9,10]. Marchese et al. [2] suggest that alpha- and gamma-tocopherol oppose each other under certain conditions. For example, some experiments show that alpha-tocopherol protects, whereas gamma-tocopherol promotes lung inflammation and airway hyper-responsiveness [10,11]. According to data from the CARDIA (coronary artery risk development in young adults) study, serum concentrations of alpha-tocopherol are favorably associated with lung function, whereas gamma-tocopherol levels are negatively related to spirometry results [2].

Other investigations focusing on the health effects of alpha- and gamma-tocopherol have also yielded conflicting findings. A meta-analysis involving approximately 370,000 participants showed that blood alpha-tocopherol levels were inversely associated with risk of prostate cancer, whereas gamma-tocopherol levels were not [12]. Further, in a NHANES (national health and nutrition examination survey) study of 1289 US adults, blood levels of alpha-tocopherol were predictive of lower fasting blood glucose concentrations, suggesting better glucose regulation, but gamma-tocopherol levels were associated with higher fasting glucose levels and also higher glycosylated haemoglobin (A1c) concentrations [13]. Additionally, in women, blood alpha-tocopherol levels were favorably associated with hemorrhagic stroke mortality, but gamma-tocopherol concentrations were directly linked to death from hemorrhagic stroke [14].

The literature includes far more investigations about alpha-tocopherol than gamma-, even though gamma-tocopherol is the primary form of vitamin E in the American diet [15]. Consequently, some researchers recommend that more research focus on gamma-tocopherol [15,16]. In a review article, Jiang et al. [16] indicate plainly that gamma-tocopherol “deserves more attention” (p. 714). Given the inconsistent findings in the literature, it is of public health interest that the biological effects of both alpha- and gamma-tocopherol be investigated and their contributions to human health be compared.

A good gauge of cell aging and biological health is the length of leukocyte telomeres. Telomeres are protective caps found on the ends of chromosomes. When cells divide, some of the telomeric DNA does not replicate. Therefore, with mitosis, telomeres become consistently shorter. Hence, it follows that scientists refer to telomeres as the molecular clock of cells [17,18].

A number of investigations show that oxidative stress shortens telomeres [19,20,21,22,23]. Furthermore, many studies indicate that shorter telomeres are predictive of premature disease, independent of age, including cancer [24,25,26], cardiovascular disease [27,28,29,30], diabetes [31,32,33], and cognitive dysfunction [34,35]. Given that oxidative stress shortens telomeres, and given the antioxidant characteristics of vitamin E and its capacity to prevent free radical reactions, it follows that vitamin E could preserve telomeres and reduce cell aging. However, little research is available indicating if vitamin E—particularly blood levels of alpha- and gamma-tocopherol—account for differences in telomere length in US men and women. Hence, the present study was conducted. A random sample of 5768 men and women collected by NHANES was used, representing non-institutionalized civilian adults in the United States. A secondary purpose was to determine the influence of several covariates, including age, gender, race, smoking, BMI (body mass index), physical activity, alcohol use, total serum cholesterol, dietary vitamin E intake, and supplement use, on the associations between alpha- and gamma-tocopherol and telomere length.

## 2. Materials and Methods

### 2.1. Sample

In order to provide national estimates of the nutrition, health, and lifestyles of individuals living in the United States, the Centers for Disease Control and Prevention administers an ongoing study called NHANES. The investigation uses a multistage probability sampling design, so the results can be generalized broadly across the United States.

Only two 2-year data cycles of NHANES contain values for telomere length, 1999–2000 and 2001–2002. All of the data are cross-sectional. The telomere data became available to the public in November, 2014, and all of the NHANES data sets are available to the public online [36].

All participants ages 20 years and older were asked to give a blood sample containing their DNA during the 1999–2000 and 2001–2002 data cycles. A total of 10,291 adults were eligible and 7827 donated a valid sample (76%). Because NHANES records the age of all participants age 85 or older as 85 to maximize confidentiality, subjects ≥85 were excluded from the sample.

To be included in the present investigation, participants were required to have data for each variable of the study. A total of 5768 adults—3043 women and 2725 men—were included. Written informed consent was obtained from each participant and the National Center for Health Statistics Ethics Review Board approved collection of the NHANES data and posting of the files online for public use [37].

### 2.2. Measures

A total of 13 variables were studied in this investigation. The exposure variables were blood levels of alpha- and gamma-tocopherol. The outcome variable was leukocyte telomere length. Covariates included: age, gender, race, BMI, smoking, physical activity, alcohol use, total serum cholesterol, supplement use, and dietary vitamin E intake.

#### 2.2.1. Alpha- and Gamma-Tocopherol

To provide objective measures of alpha- and gamma-tocopherol levels, blood specimens were collected at the NHANES mobile examination centers (MECs). Serum concentrations of alpha- and gamma-tocopherol were measured using high performance liquid chromatography (HPLC) with photodiode array detection [38]. Exclusion criteria included hemophiliacs, individuals who had received chemotherapy during the previous 4 weeks, and participants with rashes, gauze dressings, casts, edema, open sores or wounds, etc. The laboratory staff included certified medical technologists and phlebotomists. Members of the laboratory staff each completed comprehensive training before working in the MEC. The NHANES quality control and quality assurance protocols met the Clinical Laboratory Improvement Act standards [38].

#### 2.2.2. Telomere Length

NHANES [39] has described in detail the procedures employed to measure telomere length. According to NHANES, DNA was extracted from blood samples and stored at −80 °C at the Centers for Disease Control and Prevention. Specimens were then shipped to the University of California, San Francisco for analysis in the Blackburn laboratory. Leucocyte telomere length was measured using the quantitative polymerase chain reaction method and compared to standard reference DNA (T/S ratio). Five 96-well quality control plates, representing 5% of the complete set, were used. The investigators were blinded regarding the duplicate samples [39].

According to NHANES [39], “Each sample was assayed 3 times on 3 different days. The samples were assayed on duplicate wells, resulting in 6 data points. Sample plates were assayed in groups of 3 plates, and no 2 plates were grouped together more than once. Each assay plate contained 96 control wells with 8 control DNA samples. Assay runs with 8 or more invalid control wells were excluded from further analysis (<1% of runs). Control DNA values were used to normalize between-run variability. Runs with more than 4 control DNA values falling outside 2.5 standard deviations from the mean for all assay runs were excluded from further analysis (<6% of runs). For each sample, any potential outliers were identified and excluded from the calculations (<2% of samples). The mean and standard deviation of the T/S ratio were then calculated normally. The interassay coefficient of variation was 6.5%” [39]. Mean T/S ratio values were converted to base pairs using the formula: 3274 + 2413 × (T/S).

#### 2.2.3. Covariates

Age, gender, and race were used as demographic covariates. NHANES used five categories to differentiate among races and ethnicities: Non-Hispanic White, Non-Hispanic Black, Mexican American, Other race or multi-racial, and Other Hispanic. Additional covariates were employed to index lifestyle variables, including BMI, smoking, physical activity, alcohol use, dietary intake of vitamin E, supplement use, and total serum cholesterol.

#### 2.2.4. Body Mass Index (BMI)

BMI was used to compare the body weight of participants, independent of height. The standard BMI formula was used (kg/m^2^): weight in kilograms divided by height in meters, squared. BMI categories were used to differentiate among participants who were underweight (<18.5), normal weight (≥18.5 and <25.0), overweight (≥25.0 and <30.0), obese (≥30.0), or missing.

#### 2.2.5. Smoking

Pack-years, representing cumulative exposure to tobacco smoke, were used to index cigarette smoking. Pack years were calculated as the number of years smoked times the average number of cigarettes smoked per day, divided by 20.

#### 2.2.6. Physical Activity

Daily physical activity was assessed using four descriptive statements. Participants were asked to identify the statement that best described their physical activity level. Subjects were categorized according to the level of activity reported. The statements were: (1) You sit during the day and do not walk about very much; (2) You stand or walk about much of the day, but do not have to carry or lift things very often; (3) You lift light loads or have to climb stairs or hills often; (4) You do heavy work or carry heavy loads.

#### 2.2.7. Alcohol Use

A total of three categories were used to differentiate among participants relative to alcohol use: abstainers, moderate drinkers, and heavy drinkers. Heavy drinkers were women who reported drinking two or more alcoholic beverages per day over the past 12 months or men reporting three or more alcoholic drinks per day over the past 12 months. Moderate drinkers were women who reported drinking more than zero but less than two drinks per day over the past 12 months, or men who reported drinking more than zero and less than three alcoholic beverages per day during the past 12 months. Abstainers were those reporting no alcohol use in the past 12 months.

#### 2.2.8. Dietary Vitamin E Intake and Supplement Use

Using a computer-assisted interview system, a 24-h dietary recall was administered by NHANES [40]. Dietary supplement use was also assessed. Each interviewer was a college graduate in Food and Nutrition or Home Economics, with at least 10 credits in food and nutrition. Interviewers were trained and bilingual, and interviews were administered in a private setting in a NHANES MEC. The dietary assessment was used to collect detailed information about all foods and beverages consumed. Interviewers followed scripts provided in the system, and the computer-assisted program provided a standardized interview format. The diet recall included food probes that have been used in previous NHANES and USDA surveys. A multi-pass format was used during the interview. Nutrients and non-nutrient food components, including vitamin E intake, were calculated from foods and beverages that were eaten during a 24-h period prior to the interview (midnight to midnight).

#### 2.2.9. Total Serum Cholesterol

Cholesterol was measured enzymatically in a series of coupled reactions that hydrolyzed cholesteryl esters and oxidized the 3-OH group of cholesterol. One of the reaction byproducts, H_2_O_2_, was measured quantitatively in a peroxidase-catalyzed reaction that produced a color. Absorbance was measured at 500 nm. The color intensity was proportional to the cholesterol concentration [41].

### 2.3. Statistical Analysis

Strata, clusters, and individual-level sample weights were used to produce results generalizable to the non-institutionalized, civilian, adult population of the United States. By using unequal selection probability, the weights resulted in unbiased national estimates. For categorical variables, SAS SurveyFreq was used to calculate weighted frequencies to describe the data. For continuous variables, weighted means (±SE) were generated using SAS SurveyMeans. For the present study, sample weights were based on 4 years of MEC data, which included the blood alpha- and gamma-tocopherol levels and telomere lengths.

For this investigation, blood levels of alpha- and gamma-tocopherol served as the exposure variables, reported in µg/dL. The outcome variable was leukocyte telomere length. Regression analysis using the SAS SurveyReg procedure was employed to evaluate the extent of the linear associations between alpha- and gamma-tocopherol and telomere length. Regression estimates for each model were based on the complex, multistage, probability sampling process of NHANES. The effect of three demographic variables (age, gender and race) on the tocopherol and telomere relationships was tested using partial correlation and the SAS SurveyReg procedure. Additional potential mediating variables, including BMI, smoking, physical activity, alcohol use, total serum cholesterol, dietary vitamin E intake, and supplement use, were also evaluated. To study the associations between the covariates and telomere length, mean differences in telomere length were compared across each level of the covariates.

Because individuals with short telomeres are at greater risk of several diseases, the extent to which adults divided into sex-specific blood alpha- and gamma-tocopherol quartiles differed in odds of possessing short telomeres was calculated using SAS SurveyLogistic. Short telomeres were operationalized as participants in the lowest sex-specific quartile of the sample.

Statistical significance was accepted when alpha was <0.05 and all *p*-values were two-sided. SAS version 9.4 was used to conduct the statistical analyses (SAS Institute, Inc., Cary, NC, USA).

## 3. Results

In the present investigation, sample weights were employed to produce results that are generalizable to the non-institutionalized civilian adult population of the United States. Mean (±SE) age was 46.5 ± 0.5 years, and average telomere length was 5839 ± 41 base pairs. Mean blood concentrations of alpha- and gamma-tocopherol were 1328.3 ± 15.5 µg/dL and 241.5 ± 4.7 µg/dL, respectively—a 5.5-fold differential. Average dietary vitamin E intake, excluding supplements, was 7.5 ± 0.1 mg per day. Table 1 displays the weighted percentiles (±SE) for blood alpha- and gamma-tocopherol levels (µg/dL) and telomere length (base pairs), which represent those of the US adult population.

### 3.1. Age and Telomeres

In the present sample, chronological age was linearly and inversely associated with telomere length, as expected. For each one-year increase in age, telomeres were 15.6 base pairs shorter, on average (*F* = 410.4, *p* < 0.0001). Age-squared (age^2^) was not predictive of telomere length beyond the linear term (*F* = 0.3, *p* = 0.5785). Table 2 shows mean differences in the length of telomeres across sequential categories based on chronological age.

### 3.2. Gamma-Tocopherol and Telomere Length

Blood levels of gamma-tocopherol and telomere length were inversely related, as shown in Table 3. After adjusting for differences in the demographic covariates, for each µg/dL higher level of gamma-tocopherol, telomeres were 0.33 base pairs shorter, on average (*F* = 7.1, *p* = 0.0126). Telomeres were 0.27 base pairs shorter for each µg/dL increment in gamma-tocopherol with all of the covariates controlled, including age, gender, race, BMI, physical activity, smoking, alcohol use, total serum cholesterol, supplement use, and dietary vitamin E intake.

With chronologic age and telomere length both treated as continuous variables, the relationship between age and telomere length was 15.6 base pairs per year of age. Hence, telomeres were approximately 1 year shorter (15.6 base pairs) for each increment of 47.3 to 55.7 µg/dL of gamma-tocopherol in the blood, depending on the variables controlled. Given adults at the 75th percentile (quartile 3) had 163 µg/dL higher levels of blood gamma-tocopherol than those at the 25th percentile (quartile 1), as shown in Table 1, adults of the same age, gender, race, etc. at the 75th percentile had 2.8–3.4 years greater cellular aging than those at the 25th percentile, depending on the covariates controlled (163 × 0.27 = 44.0 ÷ 15.6 = 2.8 years; 163 × 0.33 = 53.8 ÷ 15.6 = 3.4 years).

### 3.3. Alpha-Tocopherol and Telomere Length

Blood levels of alpha-tocopherol were not associated with telomere length, as shown in Table 3. In general, telomeres were longer as blood alpha-tocopherol levels increased, but none of the relationships were statistically significant.

### 3.4. Alpha- and Gamma-Tocopherol

Blood levels of alpha- and gamma-tocopherol were inversely associated (*F* = 39.9, *p* < 0.0001). After adjusting for differences in the demographic and lifestyle covariates, for each µg/dL increase of gamma-tocopherol in the blood, alpha-tocopherol levels decreased by 1.2 µg/dL.

### 3.5. Dietary Vitamin E

Dietary vitamin E consumption was not related to telomere length after controlling for the demographic (*F* = 0.5, *p* = 0.4696) or the demographic and lifestyle covariates together (*F* = 0.3, *p* = 0.5977). However, vitamin E intake was inversely related to gamma-tocopherol blood levels, with the demographic covariates (*F* = 8.3, *p* = 0.0074) and the demographic plus lifestyle covariates controlled (*F* = 8.9, *p* = 0.0059). Specifically, for each mg of dietary vitamin E consumed in the diet, blood gamma- levels decreased by 1.5 and 1.2 µg/dL, respectively. Additionally, dietary vitamin E intake was directly associated with alpha-tocopherol blood levels, with the demographic covariates controlled (*F* = 12.3, *p* = 0.0015) and also with the demographic and lifestyle covariates held constant (*F* = 12.2, *p* = 0.0016). Specifically, for each mg of dietary vitamin E consumed, alpha-tocopherol blood levels were 9.2 and 9.1 µg/dL higher, respectively.

### 3.6. Dietary Supplement Use

A total of 51 ± 1% of the sample reported using dietary supplements. Use of dietary supplements was strongly related to blood levels of gamma-tocopherol. Specifically, after controlling for differences in age, gender, and race, participants who used dietary supplements (192.4 ± 6.8 µg/dL) had significantly lower gamma-tocopherol levels than those reporting they did not take supplements (279.3 ± 6.2 µg/dL)—a difference of 86.9 ± 6.1 µg/dL (*F* = 203.8, *p* < 0.0001). The relationship remained virtually identical after adjusting for differences in the demographic and lifestyle covariates combined (*F* = 204.0, *p* < 0.0001). Conversely, dietary supplement users (1569.8 ± 24.0 µg/dL) had significantly higher levels of blood alpha-tocopherol than non-supplement users (1157.7 ± 18.1 µg/dL)—a difference of 412 ± 23.4 µg/dL (*F* = 309.8, *p* < 0.0001), after adjusting for the demographic covariates. Controlling for the demographic and lifestyle covariates together produced a blood alpha-tocopherol difference of 394.9 ± 20.5 µg/dL (*F* = 371.5, *p* < 0.0001).

Dietary supplement users (7.3 ± 0.1 mg) also had higher intakes of dietary vitamin E from foods compared to non-users (6.6 ± 0.2 mg)—a difference of 0.7 ± 0.2 mg (*F* = 11.7, *p* = 0.0019), with the demographic variables controlled statistically. Comparable results were revealed after adjusting for the demographic and lifestyle covariates together (*F* = 8.3, *p* = 0.0074).

The relationship between dietary supplement use and telomere length was not significant after adjusting for the demographic covariates (*F* = 2.6, *p* = 0.1151) or after controlling for the demographic and lifestyle covariates combined (*F* = 1.3, *p* = 0.2573).

### 3.7. Odds of Possessing Short Telomeres

Participants with short telomeres (i.e., lowest sex-specific quartile) were compared to all other adults regarding their blood gamma-tocopherol levels. Specifically, participants with high levels of gamma- (i.e., highest sex-specific quartile) were compared to those in the other three quartiles. Findings showed that adults with high gamma-tocopherol levels were 53% (95% Confident Interval (CI): 1.1–2.1) more likely to possess short telomeres than their counterparts. After adjusting for the demographic covariates, the odds of having short telomeres was 46% (95% CI: 1.1–2.0) greater in those with high blood gamma- levels compared to all others. After controlling for all of the covariates, demographic, and lifestyle, the odds of possessing short telomeres was 42% (95% CI: 1.0–2.0) greater in those with high gamma- levels compared to adults with other gamma-tocopherol blood levels.

## 4. Discussion

The chief objective of the present study was to evaluate the association between blood levels of alpha- and gamma-tocopherol and the length of leukocyte telomeres—a biological marker of cellular aging—in a large nationally-representative sample of US adults aged 20–84. Results showed that blood levels of alpha-tocopherol were not related to telomere length. However, telomeres were progressively shorter as the gamma-tocopherol concentrations in the blood increased (Table 3). Moreover, adults with high levels of gamma-tocopherol (highest sex-specific quartile) were 42% to 53% more likely to possess short telomeres (lowest sex-specific quartile) compared to other adults, indicating increased risk of advanced cellular aging.

In the present investigation, telomere shortening occurred at the rate of 15.6 base pairs per year of chronologic age. In other words, 42-year-olds tended to have telomeres that were 31.2 base pairs shorter than 40-year-olds, and 75-year-olds had telomeres that were approximately 46.8 base pairs shorter than 72-year-olds. Given there was a gamma-tocopherol difference of 163 µg/dL between those at the 75th percentile compared to those at the 25th percentile, regression results revealed an estimated difference in biological age of about 3 years, based on telomere length. Similarly, by comparing those with gamma-tocopherol levels at the 10th percentile (86.7 µg/dL) to those at the 90th percentile (408.1 µg/dL), there was a cellular aging difference of approximately 6 years, depending on the variables controlled. Hence, the inverse relationship between blood gamma-tocopherol levels and telomere length appears significant and meaningful.

Insight can be gained by comparing the associations between blood gamma-tocopherol levels and telomere length to telomere length and other factors. For instance, in the present investigation, after adjusting for differences in the demographic covariates, U.S. men and women with a history of 20 pack-years of smoking had telomeres that were approximately 70.5 base pairs shorter than non-smokers (*F* = 17.8, *p* = 0.0002), indicating 4½ years of increased biological aging. Moreover, in the present sample, telomeres differed significantly in length across the various BMI categories (*F* = 3.3, *p* = 0.0246), with obese adults exhibiting telomeres that were 114.9 base pairs shorter than normal weight adults, suggesting more than 7 years of additional cellular aging. Conversely, the NHANES data showed no relationship between total serum cholesterol and telomere length (*F* = 2.4, *p* = 0.1321). Overall, it appears that blood levels of gamma-tocopherol account for differences in telomere length in US adults, consistent with common risk factors, including smoking and obesity.

According to the results, about one-half of American adults report using dietary supplements, but supplement use was not related to telomere length in this national sample. However, blood alpha- and gamma-tocopherol levels were highly related to supplement use, in opposite directions. Supplement users had 36% higher levels of blood alpha-tocopherol and 31% lower levels of gamma-tocopherol, on average, compared to their counterparts. Dietary vitamin E intake, alpha-tocopherol based, was directly related to higher levels of blood alpha-tocopherol levels and inversely related to gamma-tocopherol concentrations. Although differences in supplement use and dietary vitamin E intake were highly related to tocopherol blood levels, when statistical adjustments were made for differences in these covariates, the inverse relationship between gamma-tocopherol and telomere length remained statistically significant and the direct association between alpha-tocopherol and telomere length remained non-significant.

An extensive review by Wolf explains how the consumption of alpha-tocopherol can influence gamma-tocopherol levels [42]. The review states that excess alpha-tocopherol taken in supplements causes a reduction of gamma-tocopherol concentrations. Furthermore, reductions in gamma-tocopherol concentrations during increased consumption of alpha-tocopherol occurs because of accelerated metabolism of gamma-tocopherol. Recent research with rodents suggests that competition for an intestinal transporter protein could play a role in the reduction of gamma- concentrations associated with high doses of alpha-tocopherol [43]. The unfavorable association between gamma-tocopherol and telomere length could be partly a function of the interaction and competition between alpha- and gamma-tocopherol.

The literature includes a number of studies designed to determine the effect of supplemental vitamin E intake on disease. For example, in a classic randomized controlled trial [44], male smokers supplemented with alpha-tocopherol had no improvements in lung cancer rates, but deaths from ischemic heart disease and stroke were reduced. On the other hand, deaths from cancers other than lung cancer were higher among vitamin E users, as were deaths from hemorrhagic stroke. The authors concluded that vitamin E “may actually have harmful as well as beneficial effects” (p. 1029).

The Heart Outcomes Prevention Evaluation (HOPE) Study, which investigated nearly 10,000 adults at high risk of heart attack or stroke for 4.5 years, determined that 400 IU of natural vitamin E supplementation had no protective effect [45]. However, 2.5 years of additional treatment with vitamin E in the HOPE–TOO trial concluded with an unexpected increase in heart failure, indicating that vitamin E supplementation was unhealthy [46].

In another randomized placebo-controlled trial, no cardiovascular benefits in women with established heart disease were seen in those supplemented with vitamins E and C, but all-cause mortality was increased significantly [47]. Further, two meta-analyses of randomized controlled trials have shown that vitamin E supplementation results in increased all-cause mortality [48,49]. Consequently, none of the major health organizations in the US recommend that supplements be used to increase serum tocopherol levels.

In the present investigation, alpha- and gamma-tocopherol levels were linked to different telomere outcomes. A number of studies have shown similar conflicting results. Based on spirometric parameters, Marchese et al. [2] reported that there are opposing associations for alpha- and gamma-tocopherol. Specifically, using data from the CARDIA investigation, alpha- levels were positively related to spirometry findings and gamma-tocopherol levels were negatively related to lung-function [2]. Research by Ford et al. showed that blood glucose levels decreased as alpha-tocopherol levels increased (a desirable association), whereas fasting glucose and glycosylated haemoglobin (A1c) both increased as gamma-tocopherol concentrations increased, unfavorable outcomes [13].

Nagao et al. [14] also uncovered conflicting results in a study of more than 39,000 Japanese adults followed for more than a decade. In women, alpha-tocopherol levels were inversely related to total stroke and hemorrhagic stroke mortality. Gamma- concentrations were indirectly associated with ischemic stroke mortality in men, but directly and unfavorably related to hemorrhagic stroke in women.

Hak et al. [50] also found unhealthy results for gamma-tocopherol. Low-risk male physicians from the Physician’s Health Study were followed for up to 13 years. Those who had a heart attack were paired with controls, and a number of risk factors were adjusted statistically. After controlling for all of the potential confounders, men with high levels of gamma-tocopherol had more than twice the risk of a heart attack compared to those with low gamma- concentrations, and the overall P for trend was significant.

Overall, a number of epidemiologic investigations have produced conflicting outcomes for alpha- and gamma-tocopherol. Although both are antioxidants, their effects on health and disease are inconsistent and sometimes opposing. Findings from the present study were no exception to this pattern.

The present investigation had multiple limitations. First, because the study had a cross-sectional design, cause-and-effect conclusions are not defendable. Second, although 10 demographic and lifestyle covariates were controlled statistically, there are always other factors that could account for the undesirable relationship between gamma-tocopherol and telomere length. Similarly, residual confounding is a possibility. Elevated concentrations of gamma-tocopherol could be a marker for a generally unhealthy lifestyle, which could explain the shorter telomeres found in these adults.

There were also many strengths associated with this study. A total of 5768 adults were included in the sample, which was multi-racial and representative of the non-institutionalized population of the United States, 20–84 years of age. Second, statistical adjustments were made for several potential confounders, including age, gender, race, smoking, BMI, physical activity, alcohol use, total cholesterol, dietary vitamin E intake, and supplement use. Third, NHANES used a highly respected independent lab to measure telomere length. Fourth, chronological age was strongly associated with telomere length, consistent with the literature.

## 5. Conclusions

In conclusion, the effect of alpha- and gamma-tocopherol on morbidity and mortality appears to be erratic. In the present study, blood levels of alpha-tocopherol were not related to telomere length. However, gamma- concentrations were unfavorably and inversely linked to telomere length in a randomly selected sample of almost 6000 adults. Men and women with gamma- levels at the 75th percentile had much shorter telomeres than those at the 25th percentile, accounting for about 3 years of additional biological aging. Moreover, adults in the top quartile of gamma-tocopherol were approximately 45% more likely to possess short telomeres than other adults. Evidently, as gamma-tocopherol levels increase in the blood, cell aging increases as well. Given the many studies in the literature showing opposing findings between alpha- and gamma-tocopherol (including the present investigation), more research is clearly warranted.

## Figures and Tables

**Table 1:** Percentiles for blood levels of alpha-tocopherol and gamma-tocopherol and telomere length (base pairs) among US women and men (*n* = 5768).

Variable	Percentile (±SE)				
	5th	25th	50th	75th	95th
Alpha Tocopherol (µg/dL)					
Women (*n* = 3043)	689 ± 10	920 ± 13	1168 ± 17	1556 ± 22	2704 ± 116
Men (*n* = 2725)	678 ± 14	903 ± 9	1139 ± 23	1490 ± 31	2414 ± 59
Combined (*n* = 5768)	683 ± 10	912 ± 9	1156 ± 17	1523 ± 23	2594 ± 56
Gamma Tocopherol (µg/dL)					
Women (*n* = 3043)	65 ± 3	139 ± 5	217 ± 5	299 ± 8	486 ± 17
Men (*n* = 2725)	63 ± 4	146 ± 3	226 ± 5	311 ± 5	489 ± 15
Combined (*n* = 5768)	64 ± 3	143 ± 3	221 ± 4	306 ± 6	486 ± 14
Telomere Length (base pairs)					
Women (*n* = 3043)	4938 ± 46	5403 ± 40	5753 ± 40	6190 ± 53	7061 ± 131
Men (*n* = 2725)	4961 ± 32	5364 ± 30	5735 ± 37	6155 ± 51	7010 ± 98
Combined (*n* = 5768)	4957 ± 36	5387 ± 34	5745 ± 35	6179 ± 47	7034 ± 99

SE: standard error. Table values include person-level weighted adjustments based on the sampling design of NHANES so that values reflect those of the US adult population.

**Table 2:** Mean differences in telomere length (base pairs) across age categories in US men and women.

Variable	Age Category (Years)						*F*	*p*
	20–29	30–39	40–49	50–59	60–69	70–84		
	Mean ± SE	Mean ± SE	Mean ± SE	Mean ± SE	Mean ± SE	Mean ± SE		
Telomere Length								
Men (*n* = 2725)	6201 ± 64	5970 ± 50	5868 ± 56	5690 ± 59	5507 ± 55	5312 ± 35	136.5	0.0001
Women (*n* = 3043)	6229 ± 74	6032 ± 60	5887 ± 50	5727 ± 51	5614 ± 56	5430 ± 47	83.0	0.0001
All (*n* = 5768)	6216 ± 59	6002 ± 50	5877 ± 45	5710 ± 49	5568 ± 49	5382 ± 39	184.2	0.0001

SE: standard error. Each mean differed significantly (*p* < 0.05) from each other mean on the same row. With age and telomere length both treated as continuous variables, telomere length was 15.6 base pairs longer for each year of age (*F* = 410.4, *p* < 0.0001). Table values above include person-level weighted adjustments based on the sampling design of NHANES so that values reflect those of the US population.

**Table 3:** Relationship between gamma- and alpha-tocopherol blood levels and telomere length (base pairs) in 5768 US adults, independent of covariates.

	Telomere Length (Base Pairs)			
**Exposure**				
Variable controlled	Regression Coefficient	SE	*F*	*p*
**Blood Gamma-Tocopherol** (per µg/dL)				
demographic covariates	−0.33	0.12	7.1	0.0126
demographic and lifestyle covariates	−0.27	0.13	4.4	0.0439
**Blood Alpha-Tocopherol** (per 10 µg/dL)				
demographic covariates	0.28	0.24	1.4	0.2506
demographic and lifestyle covariates	0.36	0.34	1.1	0.3099

Demographic covariates included: age, gender, and race. The lifestyle covariates were: BMI, physical activity, pack years of smoking, alcohol use, total serum cholesterol, dietary vitamin E intake, and supplement use. Interpretation of the regression coefficients would be as follows for the first row: After adjusting for differences in the demographic covariates, for each 1 µg/dL higher level of gamma-tocopherol, telomeres were 0.33 base pairs shorter, on average. Hence, a hypothetical difference of 191 µg/dL in gamma-tocopherol would result in an estimated difference of 4 years of biological aging (191 × 0.33 = 63; 63 ÷ 15.6 = 4).
